# Disparity of Predictors for Participation in Phase 2 Cardiac Rehabilitation Between Younger and Older Patients With Cardiovascular Diseases

**DOI:** 10.7759/cureus.95281

**Published:** 2025-10-24

**Authors:** Shintaro Inoue, Taichi Ogami, Yutaro Ohnishi, Takatoshi Nishimura, Tsubasa Yokote, Ryuichi Matsukawa, Junichiro Nishi

**Affiliations:** 1 Rehabilitation, Aso Iizuka Hospital, Fukuoka, JPN; 2 Heart Failure, Aso Iizuka Hospital, Fukuoka, JPN; 3 Cardiology, Aso Iizuka Hospital, Fukuoka, JPN

**Keywords:** cart, classification and regression tree, outpatient cardiac rehabilitation, participation rate, phase 2 cardiac rehabilitation (cr2)

## Abstract

Background: Outpatient cardiac rehabilitation (OCR) is vital to improve the prognosis in patients with cardiovascular diseases (CVD). Despite its benefits, participation rates remain low, particularly among older adults. Previous studies have reported several demographic and clinical predictors for OCR participation, but differences between younger and older patients remain unclear. This study aimed to clarify disparities in predictors for OCR participation between younger and older patients with CVD.

Methods: This retrospective cohort study was conducted at a single Japanese regional core hospital from January 2019 to December 2023. Patients admitted for heart failure (HF) or ischemic heart disease (IHD) and referred to inpatient cardiac rehabilitation were included. Patients were categorized as younger (<75 years) or older (≥75 years) and further divided based on OCR participation (attended ≥1 session). Demographic, clinical, and physical function data, including the Short Physical Performance Battery and the Japanese version of the Cardiovascular Health Study criteria, were collected. Classification and regression tree (CART) analysis was used to identify predictors of OCR participation in each age group.

Results: A total of 1800 patients were analyzed (829 younger, 971 older). OCR participation rates were 21.5% in younger and 8.9% in older patients (OR 0.35, 95% CI 0.27-0.47, p<0.01). In younger patients, CART analysis identified residential address as the primary predictor, followed by left ventricular ejection fraction (LVEF) and disease type. Participation was highest (55%) in those living locally, with LVEF <47%, and admitted for IHD. In older patients, grip strength was the primary predictor, followed by residential address and LVEF. Participation was highest (58%) in those with grip strength ≥18 kg, living locally, and LVEF <26%. While LVEF and residence address were common predictors in both groups, grip strength was a unique predictor for older adults, and disease type (HF vs. IHD) was relevant only in younger patients.

Conclusions: This study revealed disparities in predictors for OCR participation between younger and older patients with CVD. Although residence address and LVEF influenced participation across both groups, grip strength and type of CVD emerged as factors in older and younger, respectively. Individual strategies are necessary to enhance participation rate in OCR and long-term patient outcomes.

## Introduction

In recent years, revascularization therapy for acute myocardial infarction and minimally invasive surgery, such as transcatheter aortic valve replacement for severe aortic stenosis, have enabled patients with heart disease to be discharged from the hospital earlier [1]. Therefore, outpatient cardiac rehabilitation (OCR) is crucial for these patients to improve their lifestyle habits and exercise tolerance after discharge. As OCR has been shown to reduce cardiovascular events [2], and participation in OCR is associated with a lower risk of all-cause mortality than non-participation [3,4], physicians and other medical staff should refer patients to participate in OCR.

However, there is a significant issue with the participation rate of patients with cardiovascular disease in OCR being notably low [5-8]. Kamiya reported that only 7.3% of patients with heart failure (HF) participated in OCR in a nationwide survey in Japan [8]. Furthermore, analogous trends were documented in both the United States and Europe [9,10]. Consequently, participating in OCR is vital for improving the prognosis of patients with cardiovascular disease.

Various predictors of whether patients participate in OCR have been revealed in previous studies [11-14], and demographic or socioeconomic factors were related to participation in OCR. Furthermore, older patients have some limitations related to these factors, such as impairment in physical function, cognitive dysfunction, and social isolation [15]. Brouwers [12] revealed that nonreferral for OCR decreased in older patients with cardiovascular diseases due to their characteristics, and other previous studies reported that they participated in OCR less than younger individuals [12,13,16]. However, older patients who were 65 years old and above can also benefit from OCR in terms of mortality [17], exercise capacity [18], and health-related quality of life [19]. In Japan, which has one of the highest aging populations in the world and a growing number of older patients with HF since 2012 [20], it is crucial to reveal the differences in predictors for participation in OCR between older and younger patients to encourage participation and provide benefits even for older individuals.

Thus, the present study aimed to investigate predictors for participation in OCR between older and younger patients with cardiovascular diseases, as few prior studies have directly compared these age groups.

## Materials and methods

Study design

This retrospective cohort study was conducted at a single center, which is a regional core hospital in Iizuka City, Fukuoka Prefecture, Japan. It is situated in the center of the Chikuho region, which consists of Iizuka City and the surrounding municipalities. Patients visit our center not only from within the city but also from all over the Chikuho area. Ethics approval was obtained from the Aso Iizuka Hospital Ethics Committee (approval number: 21077). This research was conducted with the assistance of the Aso Iizuka Hospital Clinical Research Grant.

Subjects

**Figure 1:**
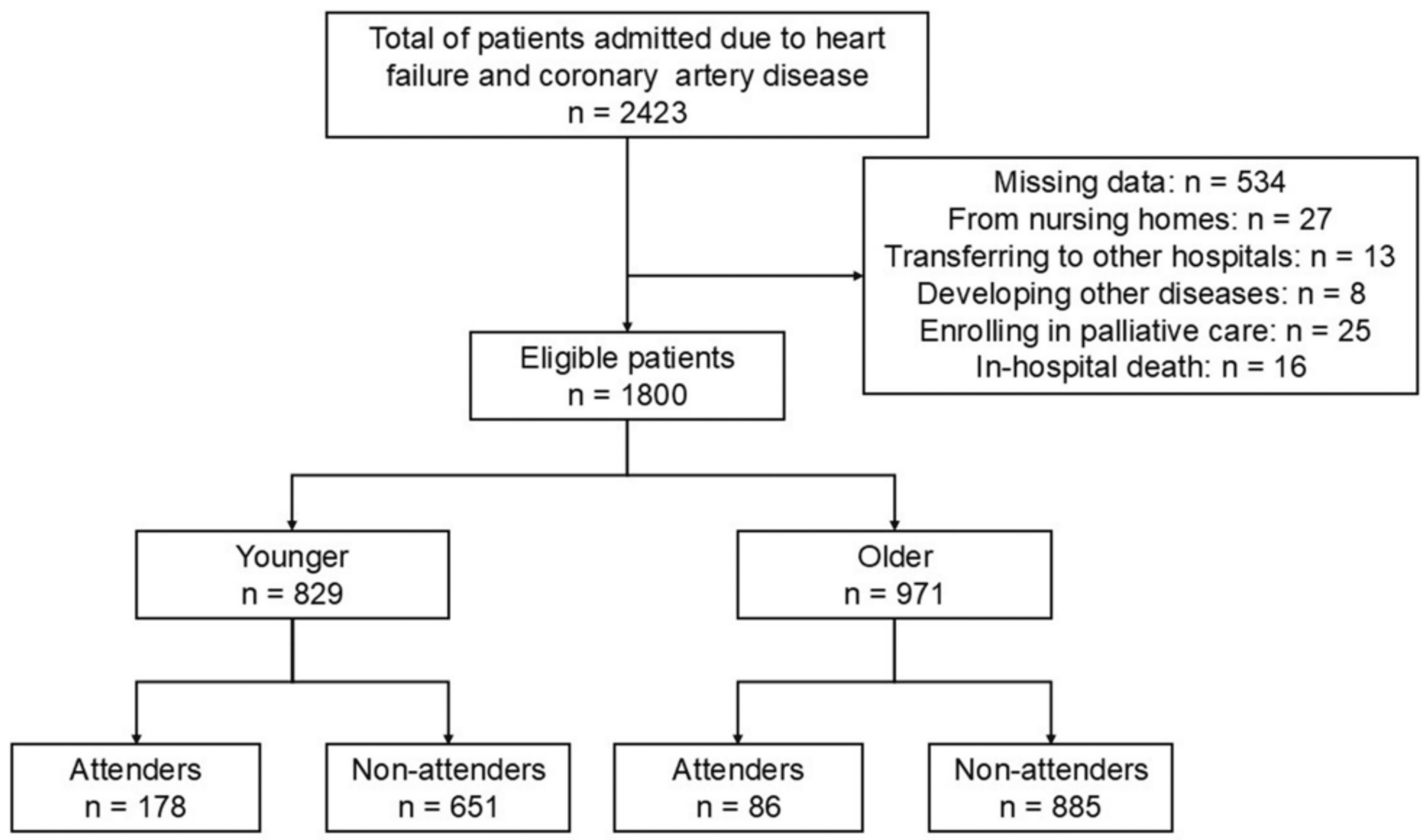
Flowchart of subjects

The subjects of the present study were patients admitted to our center due to HF or ischemic heart diseases (IHD), and inpatients OCR from January 1, 2019, to December 31, 2023. Exclusion criteria were classified as missing data, admission from the nursing care home, developing other diseases during admission, enrolling in palliative care, cases of in-hospital death, or transferring to the other hospital (Figure 1). Thus, we adopted a complete case analysis.

Cardiac rehabilitation in our center

All hospitalized patients of the cardiology department without cognitive problems or disabilities in daily life are referred to as outpatient OCR by physicians or physical therapists. Program details are explained directly to patients and, when necessary, to family members or caregivers. If the patients can visit our hospital and agree to participate in OCR, they will start OCR. Our OCR team consists of physicians, nurses, physiotherapists, clinical laboratory technicians, and dietitians. OCR participants are provided with lifestyle and exercise instructions from the OCR team members. In addition, the exercise program consists of resistance training and aerobic exercise, and the intensity was determined by their cardiopulmonary test results. These programs are performed for 60 minutes a day and once or twice a week. The standard duration of the program is 12 weeks; however, patients who have not achieved their goals or wish to continue may extend their participation.

Measurements

Demographic and clinical characteristics and physical function were collected retrospectively from medical records. Demographic and clinical characteristics included age, gender, live-in status (living with others or not), absence of long-term care insurance, employment status, residential address (local or non-local), smoking history, coronary risk factors, left ventricular ejection fraction (LVEF), and body mass index (BMI). The residential address was categorized into local and non-local. Local was defined as residence within Iizuka City, generally allowing access to our hospital within approximately 20 minutes by transportation. Non-local refers to residence in other municipalities of the Chikuho region, which typically requires longer travel times. Physical function was assessed by the physiotherapist when each patient was allowed to join the inpatient OCR program after completing the early mobilization programs, which consisted of progressive activities such as sitting, standing, and walking, performed under careful monitoring of vital signs and any signs of worsening HF. Physical functions were assessed using the Short Physical Performance Battery (SPPB), grip strength, and the Japanese version of the Cardiovascular Health Study (J-CHS) by physiotherapists. SPPB includes assessment of a standing balance test, a 4-meter walk test, and a chair sit-stand test. For the balance test, participants were asked to stand in a side-by-side position, a semi-tandem position, and a full-tandem position. In all positions, they were to hold for at least 10 seconds. For the 4-meter walk, participants were instructed to perform at a comfortable walking speed, and their walking time was measured. For the chair sit-stand test, participants were instructed to complete five chair sit-stand cycles as rapidly as possible with their arms folded across the chest and were counted from the initial sitting position to the final standing position at the end of the fifth stand [21]. Grip strength was assessed by both hands with a grip strength dynamometer (Grip-D, Takei, Japan). The stronger hand was defined as their grip strength. The J-CHS evaluates each of the following five items: grip strength assessed by grip strength dynamometer (Grip-D, Takei, Japan), walking speed, weight loss, fatigue, and physical activity. Participants who meet three or more items are defined as frail, those who meet one or two items are defined as pre-frail, and those who meet none of the items are defined as robust [22].

Outcome

The outcome of the present study was defined as participation in OCR, which involved attending one or more sessions.

Statistical analysis

Eligible patients were divided into younger and older groups. Older patients were defined as 75 and older in the present study. In line with the consensus of the Japan Gerontological Society and the Japan Geriatrics Society, which jointly in 2017 redefined older adults as those aged 75 years and above, we categorized patients into younger (<75 years) and older (≥75 years) groups. This age threshold has been widely adopted in a recent Japanese clinical study [23]. Furthermore, they were divided into attenders and non-attenders in OCR. In these groups, continuous and categorical variables were compared using the Mann-Whitney U test because the continuous variables did not follow a normal distribution, and chi-square tests, respectively. Statistical significance was applied as p-value <0.05 in the present study. To reveal the predictors of participation in OCR, decision trees were created in younger and older patients. Classification and regression tree (CART) analysis was conducted to create decision trees. In contrast to logistic regression analysis, which is a classification model, CART partitions the observed data into conditional branches and makes predictions. This approach is advantageous because it can accommodate complex and non-linear relationships between variables, which is suitable for exploring predictors of OCR participation in our cohort. Factors for which the p-value was below 0.05 in univariable analysis were included for CART analysis, and the Gini index was applied as the criterion for splits. The tuning parameter was set at 0.0001, the maximum depth was set at 3, the minimum number of cases before analysis was 5, and the minimum number of cases after analysis was 5. To evaluate the generalizability of the two CART models (younger and older groups), we performed stratified cross-validation by randomly splitting the dataset into a training set (70%) and a validation set (30%), while preserving the prevalence of the outcome. The decision trees were constructed using the training set, and performance was assessed in the validation set. Evaluation metrics included sensitivity, specificity, positive predictive value (PPV), and negative predictive value (NPV). All of the analysis was conducted by R ver. 4.4.2 (R Foundation for Statistical Computing, Vienna, Austria). The rpart package was used for constructing the decision trees, and the caret package was applied for cross-validation procedures.

## Results

Overview of the present study

In total, 2423 patients were included, and 1800 were eligible for analysis (Figure 1).

A total of 264 (14.7%) participated in OCR. Furthermore, the participation rate was significantly lower in older compared with younger patients (21.5% vs. 8.9%, odds ratio 0.35, 95% CI 0.27-0.47, p<0.01) (Table 1).

**Table 1:** Comparison of characteristics Values are represented as median (min, max), n (%). BMI: body mass index; HT: hypertension; CKD: chronic kidney disease; DM: diabetes mellitus; DL: dyslipidemia; LVEF: left ventricular ejection fraction; SPPB: Short Physical Performance Battery; J-CHS: Japanese version of Cardiovascular Health Study.

	Younger (age <75, n = 829)			Older (age ≥75, n = 971)		
	Non-participated (n = 651)	Participated (n = 178)	p-Value	Non-participated (n = 885)	Participated (n = 86)	p-Value
Age, years	66 (35, 74)	65 (28, 74)	0.32	83 (75, 101)	80 (75, 91)	<0.01
Sex, male (%)	506 (77.7)	137 (77.0)	0.84	437 (49.4)	57 (66.3)	<0.01
Disease (%)			0.04			0.03
Heart failure	371 (57.0)	86 (48.3)		674 (76.2)	56 (65.1)	
Ischemic heart disease	280 (43.0)	92 (51.7)		211 (23.8)	30 (34.9)	
Length of stay, days	11 (1, 346)	11 (4, 104)	<0.01	13 (2, 84)	13 (3, 53)	0.85
Living-in status, together (%)	489 (75.1)	134 (75.3)	1.00	638 (72.1)	71 (82.6)	0.04
long-term care insurance, yes (%)	50 (7.7)	5 (2.8)	0.02	308 (34.7)	16 (18.6)	<0.01
Smoke, current (%)	269 (41.3)	65 (36.5)	0.26	84 (9.5)	7 (8.1)	0.85
Employment status, working (%)	277 (42.5)	98 (55.1)	<0.01	22 (2.5)	11 (12.8)	<0.01
Address, non-local (%)	434 (66.7)	95 (53.4)	<0.01	524 (59.2)	28 (32.6)	<0.01
BMI, kg/m^2^	24.0 (13.8, 58.1)	24.0 (12.3, 44.4)	0.66	22.0 (13.2, 56.8)	22.3 (13.9, 31.5)	0.43
HT (%)	483 (74.2)	127 (71.3)	0.44	716 (80.9)	59 (68.6)	0.01
CKD (%)	69 (10.6)	9 (5.1)	0.03	113 (12.8)	15 (17.4)	0.24
DM (%)	262 (40.2)	68 (38.2)	0.67	315 (35.6)	35 (40.7)	0.35
DL (%)	354 (54.4)	103 (57.9)	0.44	386 (43.6)	34 (39.5)	0.50
LVEF, %	51 (12, 74)	48 (14, 74)	0.05	52 (12, 87)	48 (14, 70)	<0.01
SPPB	12 (0, 12)	12 (1, 12)	0.03	9 (0, 12)	11 (2, 12)	<0.01
Grip strength, kg	30.4 (6.5, 60.7)	32.4 (0.0, 54.4)	0.10	18.2 (0.0, 44.8)	22.60 (10.4, 45.1)	<0.01
J-CHS (%)			<0.01			<0.01
Robust	140 (21.7)	55 (31.4)		62 (7.1)	9 (10.6)	
Pre-frail	295 (45.7)	81 (46.3)		273 (31.4)	41 (48.2)	
Frail	211 (32.7)	39 (21.9)		534 (61.4)	35 (41.2)	

Younger participants

**Figure 2:**
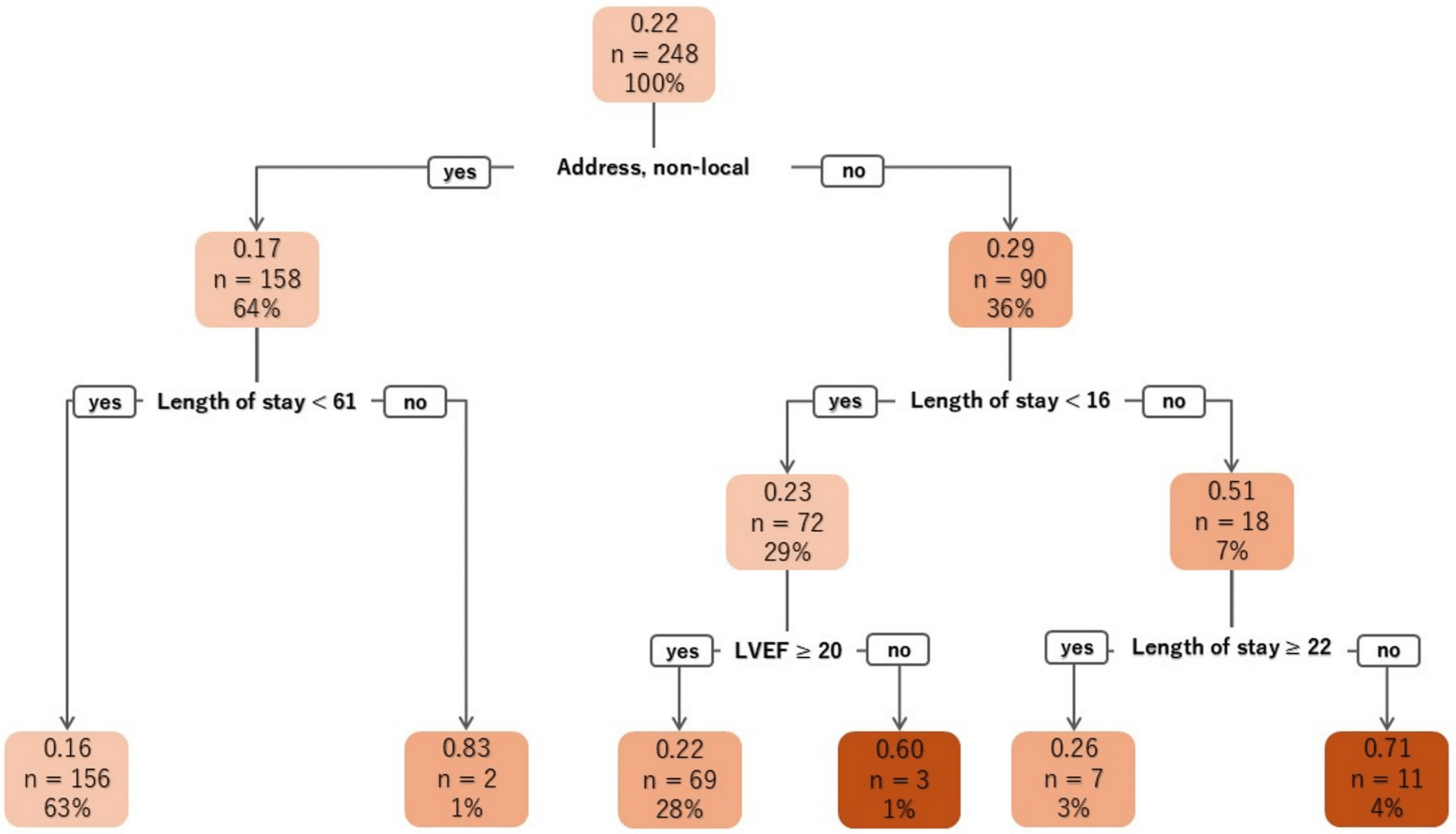
The CART of younger patients CART: classification and regression tree; LVEF: left ventricular ejection fraction.

In younger participants, the number of IHD was higher among those involved in OCR compared to non-participants. Furthermore, participants in OCR had a longer length of stay, held long-term care insurance, exhibited high rates of robustness on J-CHS, and had chronic kidney disease. They were currently employed and resided in the local area. The CART for younger individuals was created from factors including address in the first layer, length of stay in the second layer, and LVEF and length of stay in the third layer. While younger patients living non-local showed lower participation rates than the total number of younger patients, those living locally and whose length of stay ranged from 16 to 22 days had the highest participation rates in OCR, totaling 71% (Figure 2). Although the model shows that specificity was relatively high (0.94), sensitivity was extremely low (0.04), indicating a limited ability to detect participants (Table 2). 

**Table 2:** Performance metrics of CART models in younger and older participants PPV: positive predictive value; NPV: negative predictive value; CART: classification and regression tree.

Group	Sensitivity	Specificity	PPV	NPV
Younger	0.04	0.94	0.15	0.78
Older	0.08	0.99	0.67	0.92

Older participants

**Figure 3:**
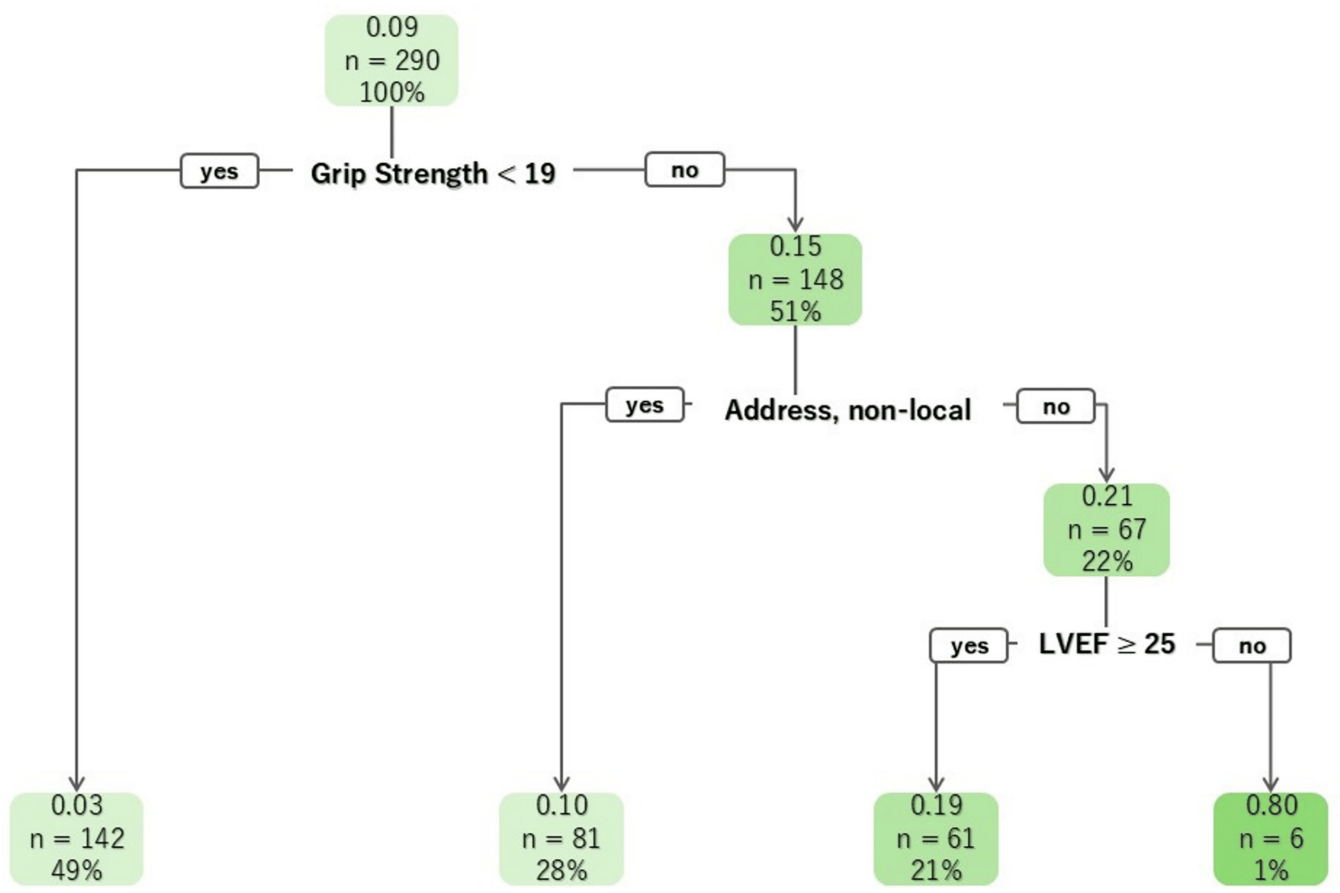
The CART of older participants LVEF: left ventricular ejection fraction.

Among the older participants, those involved in OCR exhibited a greater prevalence of IHD compared to those who did not participate. Participants in the OCR were younger than those who did not enroll, even within the older age group. They had a higher proportion of male participants, were more likely to be currently employed, and lived with their families in the local area. Additionally, they had lower rates of long-term care insurance, higher rates of hypertension, and exhibited a lower LVEF. They scored higher on the SPPB and grip strength, and were classified as robust or pre-frail on the J-CHS. The CART for older participants was created based on several factors: grip strength in the first layer, residential address in the second layer, and LVEF in the third layer. The participation rates among older patients with grip strength less than 19 kg were lower than those in the overall older patient population. Conversely, those whose grip strength was 19 kg or higher, lived locally, and had an LVEF of less than 25% showed the highest participation rates at 80% (Figure 3). Although the model shows that specificity was extremely high (0.99), sensitivity was low (0.08), as well as the model of the younger participants (Table 2).

## Discussion

The present study investigated predictors of participation in OCR among younger and older patients with cardiovascular diseases using the CART analysis. Notable disparities in the participation rates in OCR and predictors between the two groups were revealed in the present study. The overall participation rate in OCR remained low at 14.7%, with younger patients exhibiting a higher rate than older patients (21.5% vs. 8.9%). Furthermore, while LVEF and residential address were common factors, length of stay was a predictor in only younger individuals. Moreover, grip strength was derived as a predictor of participation in OCR in older patients.

LVEF

LVEF was analyzed as a factor associated with participation in OCR for both younger and older groups. Lower LVEF values were linked to higher participation rates in both age categories, with cutoff points determined as 25% for the younger patients and 27% for the older patients. The effect of exercise therapy on both patients with HF with preserved ejection fraction (HFpEF) and those with HF with reduced ejection fraction (HFrEF) was revealed in previous studies [24]. In contrast, however, mortality and readmission from cardiovascular events are observed in patients with HFrEF more than those with HFpEF [25,26]. Based on these results, as medical staff might tend to offer OCR to patients with HFrEF more frequently than those with HFpEF, patients who had lower LVEF probably showed a higher participation rate in OCR. Thus, more referrals for patients with HFpEF are necessary, as well as the education of medical staff on the benefits of OCR for not only patients with HFrEF but also those with HFpEF.

Residential area

The residence is also a crucial factor in whether patients participate in OCR [12,13,27-29], especially as both Nakayama [27] and Endo [13] revealed that the distance from home to OCR facility is a key predictor of participation in OCR among Japanese patients, as well as in the present study. Furthermore, Nakayama reported that direct and road distance were more sensitive than required time for participation rate in OCR. Our center is located in the center of our hospital region; consequently, patients who live in out-of-town areas showed a lower rate of participation in OCR than those who live in the local area. Nowadays, telerehabilitation, which was performed with remote monitoring devices, offers an accessible rehabilitation model and improves OCR adherence. Moreover, that improves the prognosis of patients with cardiovascular diseases [30-34]. However, elderly people mostly have lower digital literacy [35,36], and that gradually decreases as people get older [37]. While younger patients who live out of town have the potential to participate in telerehabilitation, older patients might not be able to do so due to the issue of digital literacy. Thus, to improve the participation rate in OCR, telerehabilitation is a better option for younger patients. In addition, other strategies for older patients, especially those who lack digital literacy, should be considered.

Older patients: grip strength

In contrast, hand grip strength was a predictor of participation in OCR only among older individuals. Muscle weakness is a predictor of the risk of cardiovascular disease and prognosis in patients with cardiovascular diseases [38-42]. Besides, low hand grip strength is related to low physical activity [43]. Patients who represent these characteristics are likely to experience exercise intolerance, fatigue, or physical limitations that can discourage participation in exercise programs. Nevertheless, older patients with cardiovascular diseases should participate in OCR, as exercise therapy improves muscle strength and positively influences the prognosis of patients with those characteristics [44,45]. In cases where they have difficulty participating in OCR, other services are necessary to provide exercise therapy, such as a home-based program.

Younger patients: length of stay

Within the CART analysis for younger patients, length of stay was identified as a factor influencing OCR participation; specifically, a shorter length of stay was associated with decreased participation in OCR. Preliminary research suggests that patients who are discharged earlier may be at risk of poor prognosis. This is due to the critical role that OCR plays in improving the prognosis of patients with cardiovascular diseases [12]. In addition, some previous studies revealed that patients with cardiovascular disease who were discharged earlier had a higher rate of readmission [46,47]. Medical staff are necessary to conduct patient education, such as the importance of medication, exercise, and diet, even in shorter lengths of stay. Moreover, referrals of OCR should also be provided by physicians, nurses, or physiotherapists more frequently.

Limitations

This study has several limitations. First, it was conducted at a single regional hospital, and the impact of residence may differ across other centers due to variations in transportation networks and referral practices; thus, multicenter studies are needed to improve generalizability. Second, the retrospective design limited the ability to control for unmeasured confounders, and some potentially important factors, such as socioeconomic status, medication use, and caregiver support, were not captured in our dataset. Third, while CART analysis was useful to identify subgroups more likely to participate, causal inference cannot be established, and some terminal nodes contained relatively small numbers of patients. Finally, external validation of the CART models was not performed, and future multicenter prospective studies are required to confirm the stability and reproducibility of our findings. Despite these limitations, a major strength of this study is the objective assessment of physical function using standardized tools and the age-stratified CART analysis, which enabled the identification of distinct predictors for younger and older patients.

## Conclusions

This study revealed important disparities in predictors for OCR participation between younger (<75 years) and older (≥75 years) patients with cardiovascular diseases. Residential area and LVEF were common predictors in both age groups. However, length of stay was a significant factor in younger patients, and grip strength was extracted as a predictor in older patients. Telerehabilitation should be implemented to improve the participation rate in OCR. Nevertheless, other interventions might be necessary because elderly patients might have lower digital literacy and physical function.
